# LINC00355 regulates p27^KIP^ expression by binding to MENIN to induce proliferation in late-stage relapse breast cancer

**DOI:** 10.1038/s41523-022-00412-2

**Published:** 2022-04-13

**Authors:** Abdallah M. Eteleeb, Prasanth K. Thunuguntla, Kyla Z. Gelev, Cynthia Y. Tang, Emily B. Rozycki, Alexander Miller, Jonathan T. Lei, Reyka G. Jayasinghe, Ha X. Dang, Nicole M. White, Jorge S. Reis-Filho, Elaine R. Mardis, Matthew J. Ellis, Li Ding, Jessica M. Silva-Fisher, Christopher A. Maher

**Affiliations:** 1 Department of Internal Medicine, Washington University School of Medicine, St. Louis, MO USA; 2 University of Missouri, Columbia, MO 65211 USA; 3 Baylor College of Medicine, Houston, TX 77030 USA; 4 The McDonnell Genome Institute, St. Louis, MO USA; 5 Alvin J. Siteman Cancer Center, Washington University School of Medicine, St. Louis, MO USA; 6 Memorial Sloan Kettering Cancer Center, New York, NY USA; 7 Institute for Genomic Medicine, Nationwide Children’s Hospital, Columbus, OH USA; 8 Department of Biomedical Engineering, Washington University School of Medicine, St. Louis, MO USA

**Keywords:** Cancer genomics, Breast cancer, Non-coding RNAs, Cell migration

## Abstract

Late-stage relapse (LSR) in patients with breast cancer (BC) occurs more than five years and up to 10 years after initial treatment and has less than 30% 5-year relative survival rate. Long non-coding RNAs (lncRNAs) play important roles in BC yet have not been studied in LSR BC. Here, we identify 1127 lncRNAs differentially expressed in LSR BC via transcriptome sequencing and analysis of 72 early-stage and 24 LSR BC patient tumors. Decreasing expression of the most up-regulated lncRNA, *LINC00355*, in BC and MCF7 long-term estrogen deprived cell lines decreases cellular invasion and proliferation. Subsequent mechanistic studies show that *LINC00355* binds to MENIN and changes occupancy at the *CDKN1B* promoter to decrease p27^Kip^. In summary, this is a key study discovering lncRNAs in LSR BC and *LINC00355* association with epigenetic regulation and proliferation in BC.

## Introduction

Breast cancer is the most common cancer diagnosed among US women with ~276,480 estimated new cancer cases in 2020 and is the second leading cause of cancer deaths among women^1^. The 5-year relative survival for localized disease is above 98%. However, this decreases significantly to 28% for distant metastasis^2^. Breast cancer relapse occurs within the first 3–5 years after initial treatment; however, those that relapse after five years are termed late-stage relapse (LSR) breast cancer^3^. Trials of patients treated with adjuvant endocrine therapy for five years have shown a 50% reduction in the risk of relapsing, but the risk of relapse is 10% and as high as 41% depending on initial tumor node status and tumor grade^4–6^. Additionally, there are limited treatment options for patients with LSR breast cancer and few of these patients cannot undergo chemotherapy owing to organ dysfunction or lower performance status as a result of widespread metastasis^7^. Overall, more studies are needed to understand the benefits of long-term hormone therapy and the underlying molecular and genetic mechanisms promoting LSR.

Long non-coding RNAs (lncRNAs) are greater than 200 nucleotides in length, do not encode proteins^8^, and have a diverse range of epigenetic and biological functions, including serving in many functions associated with carcinogenesis and metastasis^9–14^. LncRNAs have been found to be deregulated in breast cancer^15–22^ and have been associated with drug resistance^23–25^. Since lncRNAs serve as potential biomarkers due to their strong tissue specificity^26,27^, we hypothesize that they can also be used as biomarkers for LSR. Subsequent mechanistic studies could improve our understanding of why some patients relapse more than five years after treatment. However, despite multiple studies identifying the roles of lncRNAs in the distant metastasis of breast cancer^23,28–31^, the contribution of lncRNAs to LSR breast cancer has not been explored. This is primarily due to limited availability of LSR patient cohorts for molecular characterization. To overcome this barrier, we utilized ER + early-stage II and III tumor samples accrued from two neoadjuvant aromatase inhibitor treatment trials^32,33^ and LSR patient tumor samples from Washington University^34^. Using transcriptome sequencing, we compared the LSR samples to early-stage samples to identify deregulated lncRNAs associated with relapse. We identified *LINC00355* to be the most up-regulated lncRNA in LSR breast cancer patient samples and cancer cell lines. Next, we determined that *LINC00355* promoted cellular proliferation by binding to the MENIN protein to decrease the expression of cyclin-dependent kinase inhibitor, p27^Kip^. This study provides the landscape of lncRNAs in LSR and mechanistic evidence of *LINC00355* contribution in LSR breast cancer and proliferation.

## Results

### Identification of long non-coding RNAs in late-stage relapse breast cancer

**Fig. 1:**
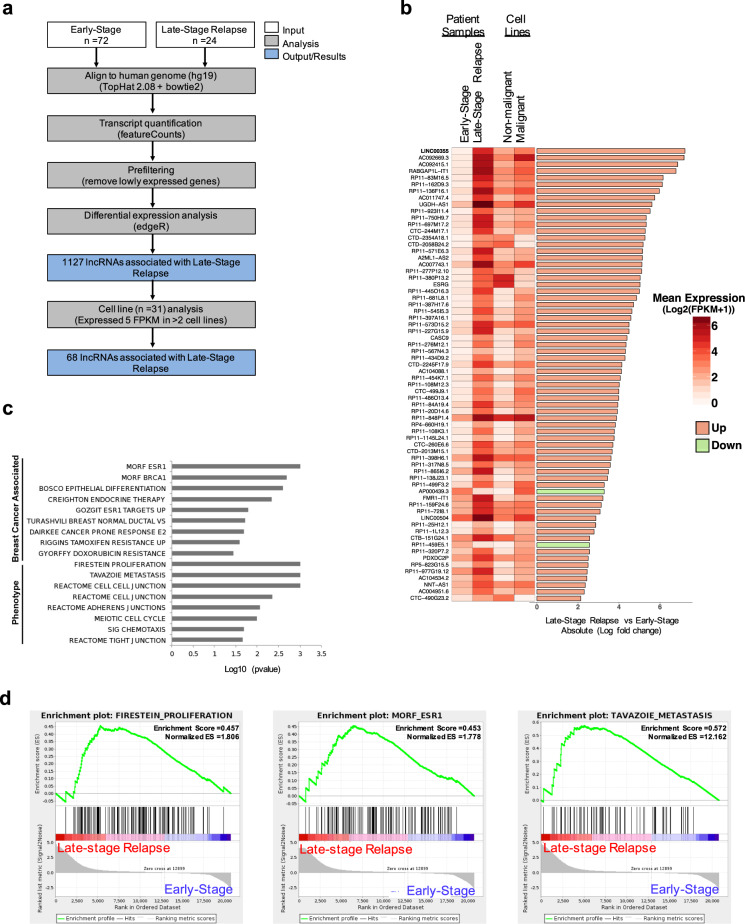
Identification of long non-coding RNAs driving late-stage relapse breast cancer*.* **a** Schematic of the pipeline used to identify lncRNAs driving late-stage relapse breast cancer. **b** Heatmap showing the mean expression of patient samples and cell lines for the lncRNAs in late-stage relapse breast cancer. Side bar represent the log fold-change. **c** Top upregulated gene sets found to be correlated with late-stage relapse as determined by GSEA. **d** Top GSEA enrichment up-regulated gene sets.

In order to identify which lncRNAs are associated with LSR, we analyzed transcriptome sequencing data from 72 early-stage patient samples from two preoperative neoadjuvant aromatase inhibitor treatment trials (ACOSOG-Z1031/NCT00265759 and NCT00084396)^32,33^, termed “early-stage” and 24 LSR patient samples sequenced at Washington University^34^, termed “late-stage” (Supp. Table 1). Our analysis revealed 1,127 differentially expressed (DE) lncRNAs (FDR < 0.001, absolute log_2_FC > 2, Supp. Table 2) between the early-stage and late-stage relapse breast cancer patient samples (Fig. 1a, Supp. Fig. 1).

Additionally, due to the complex subtypes in breast cancer, we also evaluated the expression of the DE lncRNAs associated with LSR breast cancer in a panel of publicly available RNA sequence data from 31 nonmalignant and malignant ER + breast cancer cell lines^35^. We were able to identify 68 deregulated lncRNAs associated with LSR and highly expressed in cell lines with Fragments Per Kilobase of transcript per Million mapped reads (FPKM) > 5 in at least two breast cancer cell lines (Fig. 1a, b, and Supp. Fig. 2). We identified several known lncRNAs previously identified to promote breast cancer including *LINC02582*^36^, *CASC9*^37–39^, *PDXDC2P*^40^, *NNT-AS1*^41^, *AP000439.3*^42^, and lncRNAs found to be expressed in other cancer types (Fig. 1b). Next, we performed a gene set enrichment analysis (GSEA) to evaluate the association of the identified lncRNA genes with cancer gene signatures (Fig. 1c). Our analysis found enrichment of multiple gene sets that were associated with breast cancer and estrogen receptor including MORF_ESR1 (nominal *p* value = 0.001) and GSEA sets indicating roles in FIRESTEIN_PROLIFERATION (nominal *p* value = 0.001), and TAVAZOIE_METASTASIS (nominal *p* value = 0.001, Fig. 1c and d). Using the unbiased approach of transcriptome sequencing and unique patient samples from the two clinical trials of preoperative neoadjuvant aromatase inhibitor treated and late-stage relapse patients, we identified 68 lncRNAs that we believe are associated with late-stage relapse breast cancer signaling and phenotypes.

### *LINC00355* is the most up-regulated lncRNA in LSR breast cancer

**Fig. 2:**
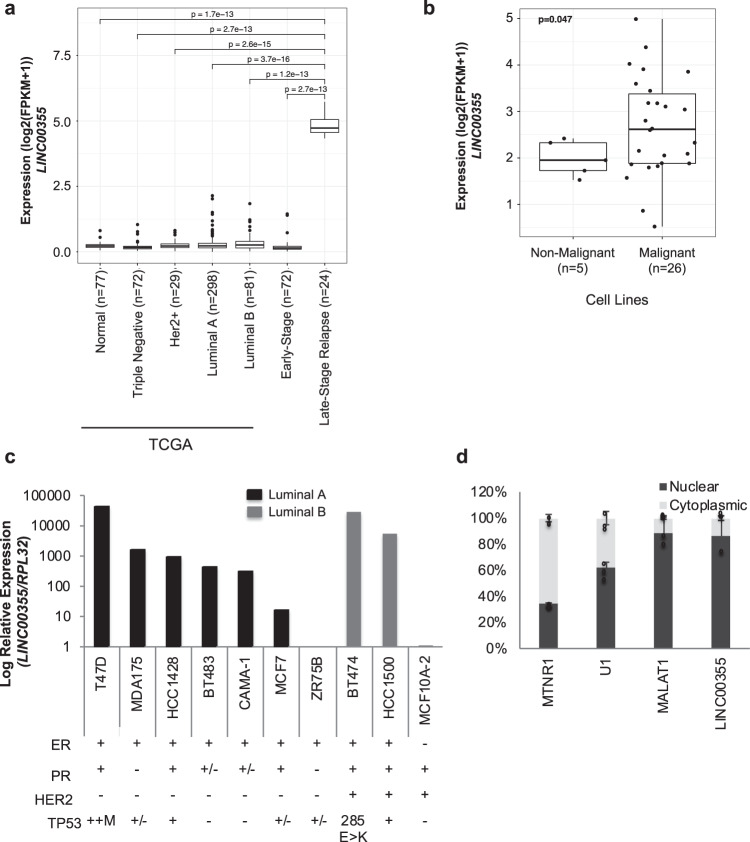
*LINC003355 characterization in breast cancer*. **a** Expression of *LINC00355* in primary tumors from the The Cancer Genome Atlas (TCGA) and early-stage and late-stage relapse from patient cohorts. **b** Sequence expression of *LINC00355* in cell line panels and **c** qPCR from in-house cell lines showing subtypes. Estrogen Receptor (ER), Progesterone receptor (PR), Human epidermal growth factor receptor 2 (HER2), Tumor protein 53 (TP53), Mutation (M) **d** Nuclear localization of *LINC00355* in T47D. MTNR1 (cytoplasmic positive control), U1, and MALAT1 (nuclear positive controls). All data are presented as mean values ± s.d., analyzed by two-tailed paired *t*-test, and repeated more than two times. Source data are provided as a Source Data File.

We focused on characterizing the top upregulated lncRNA *LINC00355* (*NR_145420.1*, Fold change= 7.21, *p* = 2.7e-13), in LSR breast cancer when comparing early-stage patient samples (Figs. 1b and 2a). *LINC00355* is a previously annotated 1878 nucleotide long lncRNA first identified as an oncogene in bladder cancer^43^. Similar to our early-stage patient cohort, *LINC00355* was downregulated in 480 early-stage breast cancer samples from The Cancer Genome Atlas (TCGA) from multiple breast cancer subtypes (Fig. 2a): triple negative (*n* = 72), mean FPKM = 0.151, HER2 + (*n* = 29), mean FPKM = 0.215, luminal A (*n* = 298), mean FPKM = 0.237, and luminal B (*n* = 81), mean FPKM = 0.299, and normal tissue (*n* = 77), mean FPKM = 0.174. *LINC00355* was only highly expressed in the late-stage relapse breast cancer patient samples (mean FPKM = 28.092, Fig. 2a). Additionally, we detected increased *LINC00355* expression in publicly available RNA-Seq data^35^ of malignant breast cell lines compared to non-malignant cell lines (*p* = 0.047, Fig. 2b, Supp. Fig. 2). To better assess *LINC00355* cell type-specific expression and show expression levels are not due to contamination of using bulk patient tissue from RNA-Seq, we also assessed its expression in single-cell RNA-Seq data from breast tissues downloaded from Wu et al.^44^ We detected very low, 0.90% or less, of cells expressing *LINC00355*, which was restricted to cancer epithelial cells (Supp. Fig. 3a–c). In contrast, *XIST* lncRNA is expressed in 35% of cancer epithelial cells and is over 50% expressed in myeloid, cancer-associated fibroblast, and endothelial cells (Supp. Fig. 3a, d, and e). We further assessed expression of *LINC00355* in normal tissue using RNA-Seq data from Genotype-Tissue Expression (GTEx) and see slightly higher expression in whole blood, omentum tissue, subcutaneous adipose tissue, and higher expression in EBV-transformed lymphocytes and testis compared to breast mammary tissue (Supp. Fig. 4). Overall, *LINC00355* is more highly expressed in LSR compared to early-stage breast cancer patient samples, and in malignant compared to non-malignant cell lines and normal tissues.

Next, we assessed *LINC00355* expression in a panel of breast cancer cell lines by quantitative PCR (qPCR) that included both luminal A and luminal B subtype cell lines. *LINC00355* is expressed greater than tenfold in breast cancer cell lines compared to the non-tumorigenic cell line MCF10-A (Fig. 2c). Since subcellular localization may provide insight into lncRNA putative functions, we fractionated estrogen receptor positive (ERα) T47D cells, which had highest endogenous levels of *LINC00355*, and found that *LINC00355* is pre-dominantly expressed in the nucleus (Fig. 2d). In summary, we show that *LINC00355* previously found to have oncogenic potential is also highly expressed in breast cancer patient samples and cell lines. Specifically, we were able to determine *LINC00355* is selectively highly expressed in LSR breast cancer.

### *LINC00355* expression promotes proliferation and invasion in malignant breast cancer cell lines

**Fig. 3:**
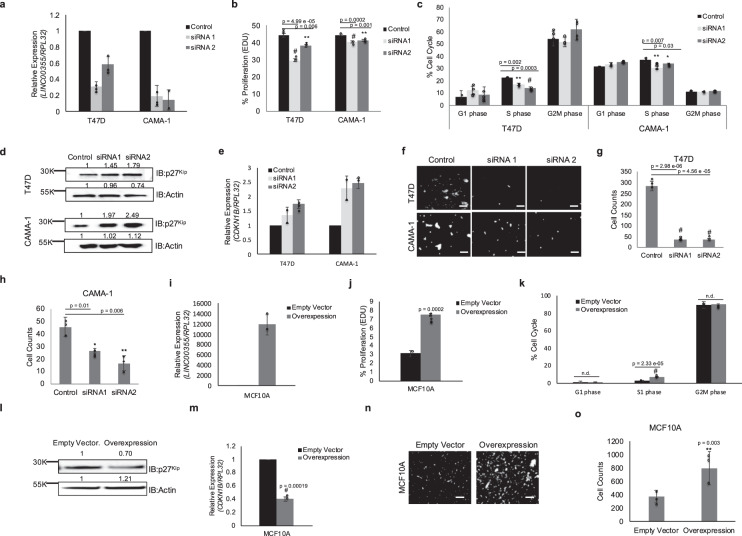
*LINC00355 induces a proliferative and aggressive phenotype in primary breast cancer cells*. **a** Transient knockdown of *LINC00355* in T47D and CAMA-1 cell lines. **b** Decreased *LINC00355* expression decreased proliferation measured by EdU incorporation and **c** decrease in S phase. Decreased *LINC00355* expression increased **d** p27^Kip^ protein and **e** *CDKN1B* mRNA levels. Fold change normalized to control. **f**–**h** Knockdown expression of *LINC00355* decreased cellular invasion. **i** MCF10A cell line with *LINC00355* overexpression and empty vector. **j** *LINC00355* overexpression increased proliferation measured by EdU incorporation and **k** increase in S phase. *LINC00355* overexpression decreased **l** p27^Kip^ protein and **m** *CDKN1B* mRNA levels. Fold change normalized to empty vector. **n** and **o** *LINC00355* overexpression increased cellular invasion. *p value < 0.05, **p value < 0.005, #*p* value < 0.0005, no difference (n.d.). All data are presented as mean values ± s.d, analyzed by two-tailed paired *t* test, and repeated more than two times. Bar = 25 µM, Dapi stained nuclei are shown in white. Source data are provided as a Source Data File.

In order to gain a better understanding of the role *LINC00355* plays in LSR breast cancer, we transiently silenced its expression with two siRNAs (siRNA1 and siRNA2, Supp. Table 3) in malignant breast cancer cell lines (T47D and CAMA-1) which have high endogenous *LINC00355* expression (Fig. 3a). As *LINC00355* was previously found to be associated with cellular proliferation^45–47^, we conducted EdU (5-ethynyl-2′-deoxyuridine) proliferation assays using flow cytometry in T47D and CAMA-1 cells with at least 50% silenced *LINC00355* expression. We observed a significant decrease of proliferation in T47D cells lines with silenced *LINC00355* compared to negative control scrambled siRNAs (siRNA1 *p* = 4.99e-05, siRNA2 *p* = 0.006, two-tailed paired *t*-test; Fig. 3b). Next, we detected a significant decrease in cell viability for 3 days post 72-h *LINC00355* knockdown compared to control siRNAs by Alamar Blue Assay (Day 5, siRNA1 *p* = 0.0007, siRNA2 *p* = 0.008; Day 6, siRNA1 *p* = 0.02, siRNA2 *p* = 0.02, two-tailed paired *t* test; Supp. Fig. 5a). In addition, we assessed DNA content in T47D cells with siRNAs targeting *LINC00355* and detected a decrease in the S phase of cell cycle (siRNA1 *p* = 0.002, siRNA2 *p* = 0.0003, two-tailed paired *t* test, Fig. 3c). A significant decrease in proliferation (siRNA1 *p* = 0.0002, siRNA2 *p* = 0.001, two-tailed paired *t* test, Fig. 3b) and S phase of cell cycle (siRNA1 *p* = 0.007, siRNA2 *p* = 0.03, two-tailed paired *t* test, Fig. 3c) was similarly seen in the second breast cancer cell line CAMA-1.

Due to the importance of the cyclin-dependent kinase inhibitor p27^Kip^ as one of the key regulators of progression from G1 to S phase in cell cycle and its frequent decreased concentration in human malignancies^48^, we confirmed protein expression of p27^Kip^ in T47D and CAMA-1 cell lines. Silencing *LINC00355* increased protein levels of p27^Kip^ in both cell lines (T47D, siRNA1 fold = 1.45, siRNA2 fold 1.79; CAMA-1 siRNA1 fold = 1.97, siRNA2 fold = 2.49, Fig. 3d). There was also more than 1.5-fold increase of *CDKN1B* mRNA, which encodes transcription for p27^Kip^ in *LINC00355* silenced cell lines (Fig. 3e).

Next, we evaluated whether *LINC00355* promotes cellular invasion by seeding Matrigel-coated transwells in a modified Boyden chamber assay. We found decreased cellular invasion when *LINC00355* is silenced in T47D cells (siRNA1 *p* = 2.98e-06, siRNA2 *p* = 4.56e-05, two-tailed paired *t* test, Fig. 3f and g) and CAMA-1 cells (siRNA1 *p* = 0.01, siRNA2 *p* = 0.006, two-tailed paired *t* test, Fig. 3f, h) compared to negative controls.

Further, we developed a MCF10A stable cell line with more than 11,500-fold *LINC00355* overexpression compared to empty vector cells that had negligible *LINC00355* expression *(*Fig. 3i). We similarly detect an increase in proliferation in overexpression cells compared to empty vector by EdU assay (*p* = 0.002, two-tailed paired t-test, Fig. 3j), Alamar Blue assay (Day 5, *p* = 0.0006; Day 6, *p* = 0.00007, two-tailed paired *t* test; Supp. Fig. 5b), and S phase in cell cycle by assessing DNA content (*p* = 2.33 e-05, two-tailed paired *t* test, Fig. 3k). There was also more than 1.5-fold decrease of p27^Kip^ protein levels (Fig. 3l) and *CDKN1B* mRNA (*p* = 0.0019, two-tailed paired *t* test, Fig. 3m) in *LINC00355* overexpressed cell lines. Finally, we found increased cellular invasion when *LINC00355* is overexpressed when compared to empty vector (*p* = 0.003, two-tailed paired *t* test, Fig. 3n and o) by modified Boyden chamber assay. These data indicate that *LINC00355* induces proliferation and invasion in cell lines possibly through the regulation of p27^Kip^.

### *LINC00355* expression promotes proliferation and invasion in long-term estrogen deprived cell lines

**Fig. 4:**
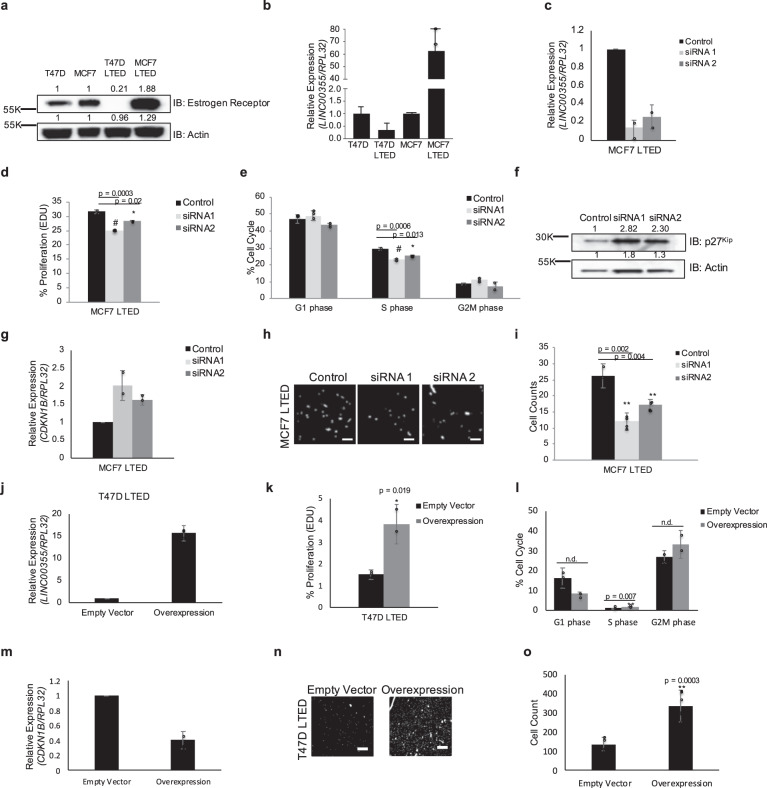
*LINC00355* induces proliferation and invasion in long-term estrogen deprived cell lines. **a** Expression of Estrogen Receptor in T47D and MCF7 wild-type and long-term estrogen deprived (LTED) cell lines. Fold change normalized to respective wild-type cell lines. **b** *LINC00355* expression in primary and LTED cell lines. **c** Transient knockdown of *LINC00355* in MCF7 LTED cell lines. **d** and **e** Decreased *LINC00355* expression in MCF7 LTED cells decreased proliferation by EdU and S phase. **f** Decreased *LINC00355* expression increased p27^Kip^ protein and **g** *CDKN1B* mRNA expression. Fold change normalized to control. **h** and **i** Cellular invasion decreased with *LINC00355* knockdown. **j** *LINC00355* expression inT47D LTED overexpression cell lines. **k** and **l** *LINC00355* overexpression in T47D LTED cells increased proliferation by EdU and S phase. **m** *LINC00355* overexpression decreased *CDKN1B* mRNA expression. **n** and **o** Cellular invasion increased with *LINC00355* overexpression. *p value < 0.05, **p value < 0.005, #*p* value < 0.0005, no difference (N.D). Bar = 25 µM. All data are presented as mean values ± s.d, analyzed by two-tailed paired *t* test, and repeated more than two times. Dapi stained nuclei are shown in white. Source data are provided as a Source Data File.

To further assess the metastatic behavior associated LSR breast cancer, we determined if *LINC00355* also induces aggressive phenotypes in a late-stage relapse setting by using two ERα + cell lines T47D and MCF7 that were deprived of estrogen for longer than 3 years, termed long-term estrogen deprived (LTED) cells^49^. The LTED model has been developed to recapitulate the acquired resistance to aromatase inhibitors. However, the two LTED models have independent mechanisms of resistance. The T47D LTED model lacks ESR1 locus amplification whereas the MCF7 LTED model has ESR1 amplification leading to increased ERα protein expression^49–51^. Wild type T47D cell lines when deprived of estrogen, termed T47D LTED, show a loss of ERα protein (Fold = 0.21) compared to its wild type counterpart (Fig. 4a). In contrast, MCF7 cell lines when deprived of estrogen, termed MCF7 LTED, show an amplified ERα protein level (Fold = 1.88) compared to its wild-type counterpart (Fig. 4a). *LINC00355* expression was decreased by 65-fold in T47D LTED when compared to wild type T47D cells as measured by qPCR, and conversely there was a 60-fold increase of *LINC00355* expression in and MCF7 LTED cell lines compared to wild type MCF7 (Fig. 4b). These results demonstrate an association of *LINC00355* with ERα.

Due to the increased levels of *LINC00355* expression in MCF7 LTED cells, we assessed whether *LINC00355* promoted aggressiveness of MCF7 LTED cells. Greater than 70% silencing of *LINC00355* in MCF7 LTED cell lines (Fig. 4c) resulted in a significant decrease of cellular proliferation (siRNA1 *p* = 0.0003, siRNA2 *p* = 0.02, two-tailed paired *t* test, Fig. 4d), Alamar Blue assay (Day 5, siRNA1 *p* = 0.015, siRNA2 *p* = 0.038; Day 6, siRNA1 p = 0.03, siRNA2 *p* = 0.0008, two-tailed paired *t* test; Supp. Fig. 5c), and S phase of cell cycle (siRNA1 *p* = 0.0006, siRNA2 *p* = 0.013, two-tailed paired *t* test, Fig. 4e) in MCF7 LTED cells compared to the negative control scrambled siRNAs. Additionally, we saw more than twofold increase in p27^Kip^ protein levels (Fig. 4f) and more than 1.5-fold increase in *CDKN1B* mRNA expression (Fig. 4g) in the MCF7 LTED cell line with silenced *LINC00355*. Further, we evaluated cellular invasion in MCF7 LTED cells lacking expression of *LINC00355*. We observed a significant decrease of invasion (siRNA1 *p* = 0.002, siRNA2 *p* = 0.004, two-tailed paired *t* test) in MCF7 LTED cells with siRNAs targeting *LINC00355* as compared to cells treated with negative control scrambled siRNA (Fig. 4h and i). Last, silenced *LINC00355* expression in MCF7 LTED cells then re-introduction of *LINC00355* expression, restores invasiveness of MCF7 LTED cells compared to silenced cells (siRNA *p* = 0.018, siRNA plus overexpression *p* = 0.012, Supp. Fig. 6).

We overexpressed *LINC00355* in the T47D LTED cell lines more than 15.5-fold compared to empty vector cells that had negligible *LINC00355* expression *(*Fig. 4j). We detect an increase in proliferation in T47D LTED overexpressed cells compared to empty vector by EdU assay (*p* = 0.019, two-tailed paired *t* test, Fig. 4k) and S phase in cell cycle by assessing DNA content (*p* = 0.007, two-tailed paired *t* test, Fig. 4l). There was also more than 60% decrease of *CDKN1B* mRNA (Fig. 4m) in *LINC00355* T47D LTED overexpressed cell lines. We lastly found an increase in cellular invasion in T47D LTED overexpressed cells when compared to empty vector (*p* = 0.0003, two-tailed paired *t* test, Fig. 4n and o). Taken together, *LINC00355* induces cellular proliferation and invasion in both malignant breast cancer cell lines and long-term estrogen-deprived cell lines that mimic late-stage relapse.

### *LINC00355* binds to MENIN to regulate *CDKN1B* expression

**Fig. 5:**
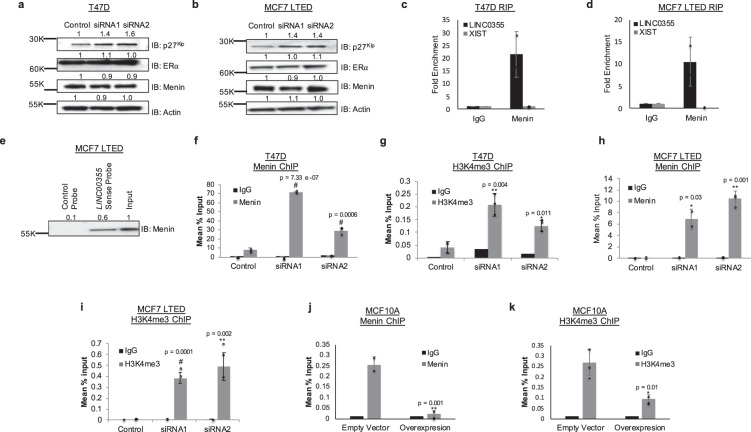
*LINC00355 binds to MENIN to regulate p27*^*KIP*^*expression*. Protein expression in **a** T47D and **b** MCF7 LTED cells upon *LINC00355* knockdown. Estrogen Receptor α (ERα). Fold change normalized to control. *LINC00355* RNA immunoprecipitation (RIP) of MENIN in **c** T47D and **d** MCF7 LTED cells. **e** MCF7 LTED RNA pull-down of *LINC00355* with MENIN. T47D cell line chromatin immunoprecipitation (ChIP) of **f** MENIN and **g** H3K4me3 at the *CDKN1B* promoter with knock down of *LINC00355*. MCF7 LTED cell line ChIP of **h** MENIN and **i** H3K4me3 at the *CDKN1B* promoter with knock down of *LINC00355*. MCF10A overexpression cell line ChIP of **j** MENIN and **k** H3K4me3 at the *CDKN1B* promoter. *p value < 0.05, **p value < 0.005, #*p* value < 0.0005. All data are presented as mean values ± s.d, analyzed by two-tailed paired *t* test, and repeated more than two times. Source data are provided as a Source Data File.

**Fig. 6:**
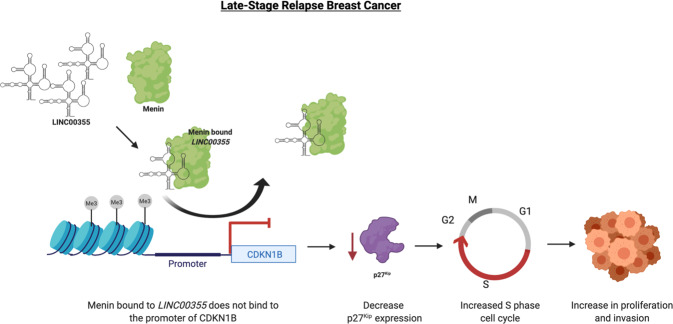
Model showing regulation of *LINC00355.* MENIN binding to *LINC00355* changes its occupancy at the *CDKN1B* promoter to decrease *CDKN1B* gene expression and p27^Kip^ protein levels. This alters S phase cell cycle checkpoint that leads to increased cellular proliferation and contributing to phenotypes of late-stage relapse breast cancer. Created with Biorender.com.

Since *LINC00355* is localized in the nucleus, increases cellular proliferation, and decreases p27^Kip^ protein levels, we hypothesize that *LINC00355* may transcriptionally regulate *CDKN1B*, the gene that encodes for p27^Kip^ protein. Previously it was shown that MENIN, encoded by MEN1 (multiple endocrine neoplasia 1), is required for its transcriptional activation of p27^Kip^ by increasing histone H3 lysine 4 methylation (H3K4me3) at the promoter of *CDKN1B*^52–55^. Thus, we assessed if *LINC00355* may directly bind to MENIN. We first assessed MENIN and ERα protein levels to show that protein levels did not change upon silencing of *LINC00355* in both the highly expressed primary cell line T47D and the LSR model MCF7 LTED cell line (Fig. 5a and b). We next conducted an RNA immunoprecipitation coupled with qPCR (RIP-qPCR) with MENIN in the T47D cell line. Indeed, we detected a more than 21-fold enrichment of *LINC00355* by RIP-qPCR compared to IgG control and do not see enrichment of *XIST* RNA, as a negative control of MENIN binding (Fig. 5c). We also show *LINC00355* increased MENIN binding in MCF7 LTED cells (Fold change = 10.4 in MENIN compared to IgG; Fig. 5d). To orthogonally validate these findings, we conducted an RNA pull-down assay utilizing a 5′ Bromo-UTP full-length *LINC00355* sense labeled probe and a negative control antisense probe to pull-down proteins that may be bound to *LINC00355*. We found that the *LINC00355* sense probe was bound to MENIN protein compared with the control probe (Fig. 5e) by Western blot of nuclear lysates. To identify the regions of *LINC00355* that bind to MENIN, we conducted cross-linking immunoprecipitation and qPCR in MCF10A cells transfected with an empty vector and *LINC00355* full length (overexpression) using eight primers tiling *LINC00355* (Supp. Fig. 7a). We detected Primer 4 and Primer 5 tiling 627-1023 nucleotides had higher fold enrichment (Fold >3 and Fold >5, respectively) of binding to MENIN (Supp. Fig. 7b). We re-validated our previous findings that full-length *LINC00355* binds to MENIN around 789 nucleotides (Fig. 5c and d). These orthogonal methods support *LINC00355* binding to MENIN protein. To determine the effects of *LINC00355*-MENIN binding at the promoter of *CDKN1B* to regulate the expression of p27^Kip^ protein, we silenced *LINC00355* to show a significant increase in MENIN occupancy at the *CDKN1B* promoter in T47D cells (siRNA1 *p* = 7.33 e -07, siRNA2 *p* = 0.0006, two-tailed paired *t* test, Fig. 5f) as compared to IgG control. We also observed an increase in H3K4me3 at the promoter of *CDKN1B* with silenced *LINC00355* (siRNA1 *p* = 0.004, siRNA2 *p* = 0.01, two-tailed paired t-test, Fig. 5g). The increase in MENIN and H3K4me3 occupancy was further detected in MCF7 LTED cells with silenced *LINC00355* in chromatin immunoprecipitation (ChIP) with MENIN (siRNA1 *p* = 0.03, siRNA2 *p* = 0.001) and ChIP with H3K4me3 (siRNA1 *p* = 0.0001, siRNA2 *p* = 0.002, two-tailed paired *t* test, Fig. 5h and i). Decreased MENIN and H3K4me3 occupancy was detected in MCF10A cells with overexpressed *LINC00355* (MENIN; *p* = 0.001, H3K4me3; *p* = 0.01, two-tailed paired *t* test, Fig. 5j and k). We additionally identified two other genes regulated by MENIN including *AGR3*^56^ and *FOXA1*^56^, that are also shown to be regulated by *LINC00355*. We show a significant decrease of expression of *CDKN1B* (*p* = 0.04), *AGR3* (*p* = 0.005) and *FOXA1* (*p* = 0.02) using a siRNA targeting *MEN1* (Supp. Fig. 8). We again validate a significant increase of *CDKN1B* expression (*p* = 0.02) with silenced *LINC00355* expression and additionally detected an increase of *AGR3* (*p* = 0.02) and *FOXA1* (*p* = 0.004) genes (Supp. Fig. 8). Taken together, we provide evidence that *LINC00355* functions by binding to the MENIN protein, which decreases its occupancy at the promoter of *CDKN1B*, decreasing protein levels of p27^Kip^, increasing proliferation, and cellular invasion in late-stage relapse breast cancer models (Fig. 6).

## Discussion

Although most relapses occur during the first 5 years after breast cancer diagnosis about 30% of ER + positive breast cancer patients relapse more than 5 years and up to 10 years after their 5-year endocrine therapy treatment. Since distant metastases are responsible for the majority of breast cancer deaths, finding ways to reduce the risk of distant metastases associated with late relapse is critical in improving survival rates from the disease. Our study used ER + early-stage (II and III) tumor tissues isolated from two neoadjuvant aromatase inhibitor (AI) therapy trials. This unique set of tumor samples allowed us to assess lncRNA expression in a preoperative AI treatment setting that is currently being assessed as a new treatment option. Additionally, we incorporated a unique very limited cohort of LSR patients. Thus, our study provides a systematic transcriptome analysis of lncRNAs expression in early-stage and late-stage relapse breast cancer patients to detect deregulated lncRNAs significantly altered in LSR breast cancer.

Through our analysis of lncRNAs in LSR breast cancer, we prioritized the most up-regulated lncRNA, *LINC00355*. Notably, *LINC00355* was previously reported to promote proliferation in multiple cancer types including bladder cancer, colorectal cancer, prostate cancer, lung adenocarcinoma, and head and neck squamous cell carcinoma^43,45–47,57–59^ and induce epithelial to mesenchymal transcription, and serve as a diagnostic biomarker in colon cancer^45–47,57–60^; however, it has not yet been identified in breast cancer and identified here specifically in ER + late-stage relapse breast cancer. High nuclear enrichment of *LINC00355* was also previously detected in bladder cancer^43^, which we confirmed in breast cancer. This led us to hypothesize that *LINC00355* may also play a role in inducing proliferation in LSR breast cancer through transcriptional regulation of key genes. Interestingly, we found that *LINC00355* induced proliferation and cellular invasion in malignant breast cancer cell lines. We also utilized MCF7 and T47D long-term estrogen receptor deprived cells lines that mimic late-stage relapse patient samples as they have been deprived of estrogen for longer than three years and have previously been shown to provide an in vitro parallel of patients treated with aromatase inhibitor having post-treatment loss or amplification of estrogen receptor^61^. When using the LTED cell lines, we showed *LINC00355* induced proliferation and cellular invasion. Because the MCF7 LTED cell line has amplified estrogen receptor protein, we have also provided evidence that *LINC00355* may have a role in an ER + amplified setting, a hypothesis that needs further investigation.

As *LINC00355* was able to induce proliferation, we decided to investigate a key cell signaling regulator, p27^Kip^. p27^Kip^ signaling is highly complex and has multiple modes of both transcriptional and post-transcriptional regulation^62^. Indeed, we found that our breast cancer cell lines with high levels of *LINC00355* also had decreased expression p27^Kip^. Moreover, we determined that *LINC00355* expression is also inversely correlated with *CDKN1B* gene expression. This indicated that *LINC00355* might transcriptionally regulate *CDKN1B* to alter cellular proliferation. We hypothesized that *LINC00355* may interact with a negative regulator of cell cycle separating the protein from its native target, thus causing an uncontrolled increase in cellular proliferation. There are numerous well-known examples of lncRNAs that function as negative regulators or decoys including: *CISAL*^63^, *ROR*^64^, *PANDA*^65^, and *MEG3*^66^. Previous research determined that the *CDKN1B* gene is transcriptionally negatively regulated by MENIN in ER + breast cancer^56^ through epigenetic modifications and maintenance of transcription at multiple loci for cell cycle regulators^52,54,67^. Given this, we focused our study on MENIN to determine if *LINC00355* interacts with MENIN sequestering it from the promoter of *CDKN1B* gene leading to decreased p27^Kip^ protein levels and disruption of the cell cycle control and increasing proliferation. This study indicates a lncRNA to bind to MENIN which provides evidence of the important functional regulation of lncRNAs. An important unanswered question is the stoichiometry of *LINC00355* in regulating the association of MENIN with the CDKN1B locus. Further, we highlight the importance of lncRNA expression in LSR breast cancer that may be used as novel therapies in the future. In conclusion, our study provides a landscape of lncRNAs in LSR and provides key evidence of their contribution to LSR breast cancer.

## Methods

### Clinical samples and cell lines

The primary breast cancer samples for this study were accrued from two neoadjuvant endocrine therapy trials^32,33^. RNA-Seq data accrued from two neoadjuvant aromatase inhibitor therapy trials (ACOSOG-Z1031, NCT00084396, and NCT00265759)^32–34^ are available via the dbGAP database (with accession phs000472). The studies were supported by the Clinical Trials Support Unit and approved by the institutional review boards of all participating institutions; all patients provided signed informed consent. Twenty-four late-stage relapse patients were enrolled and consented under a banking protocol approved by the Washington University School of Medicine Institutional Review Board (approval number 201102244, Supp. Table 1), their tumors were resected for RNA sequencing. PolyA RNA was isolated and created into Illumina TruSeq libraries run on Illumina HiSeq 2000 platform (accession GSE189389). The RNA-Seq gene expression data (FPKM) and the clinical data of TCGA BRCA samples were downloaded from the TCGA data portal. RNA-Seq data of the 32 breast cancer cell lines were obtained from the NCBI Gene Expression Omnibus (accession GSE48216)^35^. All cell lines with “unknown” subtype were removed and a representative number of cell lines from each subtype were retained.

### Sequence alignment and transcript quantification

All sequencing reads from both patient and cell lines data were aligned to the human reference genome hg19 Ensembl release 75 using TopHat version v2.0.8^68^. TopHat was run according to an in-house pipeline at The McDonnell Genome Institute (bowtie-version = 2.1.0, library-type fr-unstranded, mate-inner-dis 254, and mate-std-dev 50). The remaining TopHat parameters were left to their default values. For accurate alignment, transcriptome index file (transcript sequences) was provided to guide the alignment. Raw read counts for transcripts were generated using featureCounts version v1.4.6-p3^69^ and were used to compute transcript expression levels as normalized in FPKM (Fragments Per Kilobase of transcript per Million mapped reads) format.

### RNA-Sequencing data analysis

Differential expression analysis was performed between early-stage and late-stage relapse samples using the negative binomial generalized log-linear model and likelihood ratio tests capabilities of edgeR version v3.8.6^70^ using the raw read counts. To correct for batch effect, RUVSeq R package version v1.0.0^71^ was used with a list of negative control genes^72^. To enhance our confidence in the differentially expressed genes we discovered, lowly expressed transcripts were removed and only transcripts with expression > 1 FPKM in at least 50% of samples in either group were retained for downstream analysis. All transcripts with FDR < 0.001 and absolute log fold change > 2 were considered differentially expressed transcripts. TCGA subtypes and cell lines expression difference significance was calculated specifically for *LINC00355* using the nonparametric Wilcoxon rank-sum test.

### Gene enrichment analysis

To identify gene signatures that are associated with the identified differentially expressed genes, a gene set enrichment analysis (GSEA)^73^ was performed on the list of up and down regulated lncRNAs with 1000 gene set permutations using Signal2Noise metric. Significantly enriched gene sets were determined by false discovery rate (FDR) adjusted *P* ≤0.1 and normalized enrichment score (NES) ≥ 1.5.

### Cell Culture

All breast cancer cell lines were a kind gift from Dr. Matthew Ellis and Jieya Shao at Washington University in St. Louis. T47D, MCF7, HCC1428, BT483, ZR75B, and HCC1500 cell lines were grown in RPMI 1640 media (Invitrogen, Carlsbad, CA), 10% fetal bovine serum (Sigma, St. Louis, MO), 1% HEPES (Sigma), 0.5% glucose (Sigma), 1% sodium pyruvate (Sigma), 1% L-glutamine (Sigma), and 1% penicillin/streptomycin (Invitrogen, Carlsbad, CA). T47D LTED and MCF7 LTED cells were grown in phenol red free RPMI 1640 media (Invitrogen), 10% Charcoal stripped bovine serum (Sigma), 1% HEPES, 0.5% glucose, 1% sodium pyruvate, 1% L-glutamine, and 1% penicillin/streptomycin. CAMA-1 and BT-474 cells were grown in DMEM (Invitrogen), 10% fetal bovine serum, and 1% penicillin/streptomycin. MDA175 were grown in DMEM F12 (Invitrogen), 10% fetal bovin serum, and 1% penicillin/streptomycin. MCF10A cells were grown in DMEM F12 (Invitrogen), 10% horse serum serum, 0.5 μg/mL hydrocortisone, 10 μg/mL insulin, 20 ng/mL EGF and 1% penicillin/streptomycin. We silenced expression of *LINC00355* using custom silencer select RNAs (siRNAs) targeting *LINC00355* or Silencer Select Negative Control No.1 siRNA (Thermofisher, Austin, TX). siRNA sequences are listed in Supplementary Table 3.

Full-length *LINC00355* transcript was PCR amplified from T47D cells and cloned into the pCFG5-IEGZ vector (a kind gift from Dr. Ron Bose, Washington University). Full-length *LINC00355* inserts were confirmed with Sanger sequencing at GeneWiz. Retroviral infection of cells was performed according to Kauri et al.^74^. Briefly, the amyotrophic phoenix cell line was transfected with 10 μg of pCFG5-*LINC00355* or empty vector control by calcium phosphate precipitation and incubated for 24-h. Viral supernatants were harvested after an additional 24-h incubation. Virus was added to cells seeded in six-well dishes in the presence of 8 μg/mL polybrene (Sigma), centrifuged at 300 Å~ *g* for 90 min, and fresh media was added to the plate. After 14 days of Zeocin (Invitrogen) selection cells were used for assays. MCF10A cells or T47D LTED cells that had low endogenous expression of *LINC00355* were infected with virus expressing *LINC00355* or empty vector for 48 h and selected with 100 μg/mL Zeocin.

### Nuclear cytoplasmic isolations

Nuclear and cytoplasmic isolations were conducted using the PARIS Kit (Thermo Fisher, Waltham, MO) following the manufacturer’s protocol. Total RNA was collected as described below. Nuclear and cytoplasmic isolations were calculated by normalizing respective gene to total RNA expression.

### Quantitative real-time PCR

Total RNA was isolated for each breast cancer cell line using NucleoSpin RNA plus with DNA removal column (Macherey-Nagel, Duren). Total RNA was then transcribed to cDNA with SuperScript III First-strand cDNA system (Invitrogen) to verify expression of genes and verify knockdown efficiencies using Fast SyberGreen Master Mix (Invitrogen) as per the manufacturer’s protocol. Primer sequences are available in Supplementary Table 3.

### Modified Boyden chamber assay

Cell lines were seeded at 350,000 cells in a six-well dish. The next day cells were transfected at 50 nM with two independent custom designed siRNAs or a negative scramble control (Supplementary Table 3) with Lipofectamine RNAiMax (Invitrogen) for 72-h or 2 µg pCFG5-*LINC00355* or empty vector control with Lipofectamine 3000 (Invitrogen) for 72-h. Cells were then harvested and re-seeded in complete media at 200,000 cells on an 8.0 µM permeable membrane support transwell (Corning, Corning, NY) pre-coated with 200 µg/mL Matrigel (Corning) in 24-well plates creating a modified Boyden chamber assay. A serum gradient was established with cells plated in serum-free media added to the bottom of the well. Cells were allowed to invade overnight and then fixed with 4% paraformaldehyde (Electron Microscopy Sciences, Hatfield, PA). Next, nuclei were stained with DAPI (Sigma) (1 µg/µL). A cotton swab was used to remove non-invading cells from the top of the membrane. Invaded DAPI-stained cells were then imaged with Q-Capture Pro software on an Olympus IX70 microscope, quantified using ImageJ software (http://imagej.nih.gov/ij/), and statistical significance was determined by a student’s *t* test. Five or more images were taken per transwell membrane at 20× magnification. Assays were repeated two to three times.

### Proliferation assay

Cell lines were seeded at 350,000 cells in a six-well dish. The next day cells were transfected at 50 nM with siRNAs targeting *LINC00355* or negative control pCFG5-*LINC00355* or empty vector control as described above in Modified Boyden chamber assay section. Seventy-two hours later cells were pulsed with EdU (5-ethynyl-2′-deoxyuridine) (Thermo Fisher, Carlsbad, CA, cat# C10420) for 3 h and harvested by trypsinization. Cells were then fixed, permeabilized, and washed following manufacturer’s instructions. Cells were stained for DNA content with FxCycle Violet (Thermo). Analysis of EdU and cell cycle was assessed by measuring DNA content on a flow cytometer machine (FACScan, Becton Dickinson) at the Siteman Cancer Center Flow Cytometry Core. We collected a minimum of 25,000 cells per sample in triplicate. FlowJo Version10 (Becton Dickinson) was used to analyze data.

### Western blot

Protein was collected by plating 300,00–350,000 representative cancer cells in a six-well dish. Cells were transfected as described above. Cells were then lysed with Tris Lysis buffer (50 mM TrisHCl, 1% Triton X-100, 131 mM NaCl, 1 mM sodium orthovanadate, 10 mM Na_4_P_2_0_7_, 10 mM NaF, 1 mM EDTA, and proteasome inhibitor), run on an agarose gel and transferred to nitrocellulose membranes. Blots were then probed overnight at four degrees with respective antibodies. All antibodies and concentrations are listed in Supplementary Table 4. Blots were then washed with TBST buffer and then applied with secondary goat anti-rabbit HRP linked or goat anti-mouse HRP-linked antibodies (Thermo Fisher, Waltham, MA). Lastly, blots were washed, visualized with Clarity Western ECL Substrate (BioRad, Hercules, CA) and imaged using the ChemiDoc XRS + System (BioRad). Blots were derived from the same experiment. Raw western blots are shown in Supplementary Fig. 9 were processed in parallel and derived from the same experiment.

### RNA immunoprecipitation (RIP)

RIP coupled to qPCR assays were conducted by isolating nuclear lysates from ten million T47D or MCF7 LTED cells following the NER-PER Nuclear and Cytoplasmic Extraction Reagent (Thermo Fisher). Nuclear lysates were then incubated overnight rotating with 5 µg of Anti-Menin antibody or IgG antibody isotype control in RIPA wash buffer (50 mM Tris-HCl pH 7.4, 150 mM NaCl, 1 mM MgCl_2_, 1% NP40, 0.5% Na-Deoxycholate, 0.05% SDS, 1 mM EDTA) and SUPERase-in RNAse inhibitor (Invitrogen). The next day 50 µL of Invitrogen Dynabeads Protein G were added to the antibody lysate/mixture and rotated for 1–2 h at 4 °C. Next, beads were washed six times with RIPA wash buffer using a magnetic bead separator. Protein was then digested with Proteinase K buffer (RIPA buffer, 10% SDS, 10 mg/ml Proteinase K), at 55 °C for 30 min shaking. RNA was phenol:chloroform:isoamyl alcohol extracted following the general protocol (Thermo Fisher). Last, gDNA was removed from RNA using ArticZymes Heat and Run gDNA removal kit following the manufacturer’s protocol (Tromso, Norway). cDNA was made using SuperScript III First strand cDNA system as indicated above and qPCR was run with Fast SyberGreen MasterMix and indicated primers (Supplementary Table 3). Fold enrichment of qPCR results were calculated following Sigma-Aldrich Data Analysis Calculation Shell by comparing non-specific control IgG antibody raw CTs to MENIN or H3K4me3 RNA binding protein CT normalized against 1% input.

### BrU-labeled RNA pull-down

Full-length *LINC00355* RNA probes were made using the Promega Riboprobe in vitro transcription kit from 2.5 μg of linearized DNA in the pGEM-3Z vector (Madison, WI). Control antisense probes were made by in vitro transcription from the SP6 promoter. *LINC00355* RNA pull-down experiments were performed in MCF7 LTED nuclear lysates following the RiboTrap Kit manufacturer’s protocol (MBL, Woburn, MA).

### Chromatin immunoprecipitation (ChIP)

ChIP coupled to qPCR assays were conducted by first sonicating five million cells in SDS lysis buffer (1% SDS, 500 mM EDTA, 50 mM Tris-HCl pH8). Next, immunoprecipitation with 5 µg of IgG, MENIN, or H3K4me3 antibodies was done by incubating sonicated lysate with indicated antibody in ChIP Dilution Buffer (0.01% SDS, 1.10% Triton X-100, 1.2 nM EDTA, 16.7 mM Tris-HCl pH8, 167 mM NaCl), and 1X Halt Protease and Phosphatase inhibitors overnight with rotation at 4°. The next day 50ul of Dynabeads Protein G (Invitrogen) were added to the antibody lysate mixture and rotated for 1 h. Bead/lysate mixture was then washed once with Low Salt Wash Buffer (0.1% SDS, 1% Triton X-100, 2 mM EDTA, 20 mM Tris-HCl pH8, 150 mM NaCl), then High Salt Buffer (0.1% SDS, 1% Triton X-100, 2 mM EDTA, 20 mM Tris-HCl pH8, 500 mM NaCl), Lithium Chloride Wash Buffer (0.25 M lithium chloride, 1% NP40, 1% sodium deoxycholate, 1 mM EDTA, 10 mM Tris-HCl pH8), and finally two washes with Tris-HCl EDTA Buffer (10 mM Tris-HCl pH8, 1 mM EDTA). DNA was eluted by incubating beads for 30 min at room temperature with SDS Elution Buffer (1% SDS, 0.1 M sodium bicarbonate) followed by 1.25 M NaCl and 2.5 mg/ml RNAse A at 95° for 15 min shaking followed by addition of Proteinase K buffer (1 µL 10 mg/ml Proteinase K, 5 µM 0.5uL EDTA, 10 µL 1 M Tris pH7.5) shaking at 60° for 15 min. DNA was then isolated using phenol:chloroform:isoamyl alcohol extraction following the general protocol as mentioned above. DNA was diluted by five and used for qPCR. The % input calculation was determined by comparing CT values from input DNA and ChIP DNA for the *CDKN1B* target promoter region using the following equation: %Input = % of starting input fraction × 2^[CT(input)−CT(ChIP)]. Primer sequences are available in Supplementary Table 3.

### Single-cell RNA sequencing analysis

Breast single-cell data from 26 patients using the 10x chromium platform was downloaded from Gene Expression Omnibus (GSE176078) from Wu et al.^44^ Seurat v4.1.0 (Butler et al.^75^; Hafemeister and Satija^76^) was used for all subsequent analyses. We applied a series of quality filters to the data to remove barcodes which fell into any one of the following categories recommended by Seurat: transcript counts below 300; total genes expressed below 200 and above 10,000; UMI count below 1000 or above 10,000; mitochondrial gene expression larger than 10%. The Seurat object was constructed using the published gene and feature matrices across the sample cohort. The dataset was scaled and normalized and corrected for batch effects using Seurat’s “SCTransform” function (regressing by nCount_RNA and percent of mitochondrial DNA, variable.features *n* = 2000). Cells were clustered using the Louvain algorithm (Blondel et al.,^77^) and top 30 PCA dimensions using the following functions: “FindNeighbors” and “FindClusters” (resolution = 0.5). The resulting merged and normalized matrix was used for the subsequent analysis. Cells were annotated using the published metadata from the original publication.

### Alamar blue assay

Cell lines were seeded at 350,000 cells in a six-well dish. The next day cells were transfected at 50 nM with two independent custom designed siRNAs or a negative scramble control (Supplementary Table 3) with Lipofectamine RNAiMax (Invitrogen) for 72 h or 2 µg pCFG5-*LINC00355* overexpression or empty vector control with Lipofectamine 3000 (Invitrogen) for 72 h. Cells were then harvested and re-seeded in complete media in 96-well plates of cells. The plates were incubated for 3 days. Percent viability was scored by incubating cells for 3-h with AlamarBlue HS Cell Viability reagent (Invitrogen A50100). The reaction was stopped by the addition of 1% SDS. Fluorescence Ex/Em 540/590 was read in a Varioscan Lux plate reader. The fluorescence values for the vehicle plates were averaged and percent viability was determined by the formula: Percent viability = (average vehicle − value)/(average vehicle − average resazurin in media blank) Å~ 100.

### Cross-linking immunoprecipitation (CLIP)

Prospective cells are seeded at twenty million in 150 cm dish. The next day cells are washed with 15 ml ice cold PBS twice and adjusted to 10mls per dish. Dishes are uncovered and irradiated with 150 mJ/cm^2^ of UVA (254 nm) in Stratalinker. Cells are then harvested and centrifuged at 2000 RPM at 4 °C for 5 min. Cell pellets are resuspended by adding 1 ml of NP-40 lysis buffer (20 mM Tris–HCl at pH 7.5, 100 mM KCl, 5 mM MgCl_2_, and 0.5% NP-40) with 1ul protease inhibitors, and 1 mM DTT then incubated on ice for 10 min and centrifuge at 10,000 RPM for 15 min at 4 °C. Supernatants were collected and 1U/μl RNase T1 was added then incubated at 22 C for 30 min. 35ul of 5 M EDTA was added to stop reaction. Protein G Beads were washed two times with ice-cold PBS per sample and resuspended in 100uls NT2 buffer (50 mM Tris–HCl at pH 7.5, 150 mM NaCl,1 mM MgCl_2_, 0.05% NP-40) with 5ug of respective antibody, then subsequent rotation for 1 h at room temperature. All antibodies and concentrations are listed in Supplementary Table 4. Beads are then washed with NT2 buffer to remove excess antibody. Lysates are then added to beads for three hours at 4 °C, washed and incubated with 20 units RNAse-free DNase I for 15 min at 37 °C thermomixer shaking slowly. Protein kinase buffer (141 uls NP-40 lysis buffer, 0.1% SDS, 0.5 mg/ml Proteinase K) is then added and incubated for 15 min at 55 °C on thermomixer shaking at max speed. Supernate is then collected and isolation of RNA is conducted using standard phenol:cholorform:isoamyl alchohol protocol.

### Reporting summary

Further information on research design is available in the Nature Research Reporting Summary linked to this article.

## Supplementary information

Supplemental material merged

Supplementary Table 1

Supplementary Table 2

Supplementary Table 3

Supplementary Table 4

Reporting Summary

## Data Availability

The early-stage breast cancer RNA sequencing data referenced in this study are available from dbGaP database under the accession code phs000472 and the late-stage relapse breast cancer relapse RNA-Seq data generated in this study are available in the NCBI Gene Expression Omnibus (GEO) under the accession code GSE189389. The RNA-Seq data of the breast cancer cell lines referenced in this the study are available in a public repository from the NCBI GEO under the accession code GSE48213. The data used for the GTEx analyses described in this manuscript were obtained from the GTEx Portal (accession number phs000424.vN.pN) on 11/18/21. The source data underlying figures are provided as a Source Data file. All the other data supporting the findings of this study are available within the article, its supplementary information files, and from the corresponding author upon request. A reporting summary for this article is available as a Supplementary Information file.
