# Supplementary material for: LINC00355 regulates p27KIP expression by binding to MENIN to induce proliferation in late-stage relapse breast cancer
Source: NPJ Breast Cancer. 2022 Apr 13;8:49. doi: 10.1038/s41523-022-00412-2 (PMC9007952; doi:10.1038/s41523-022-00412-2)
Supplement: Supplementary file 1 — Supplemental material merged [file 41523_2022_412_MOESM1_ESM.pdf]

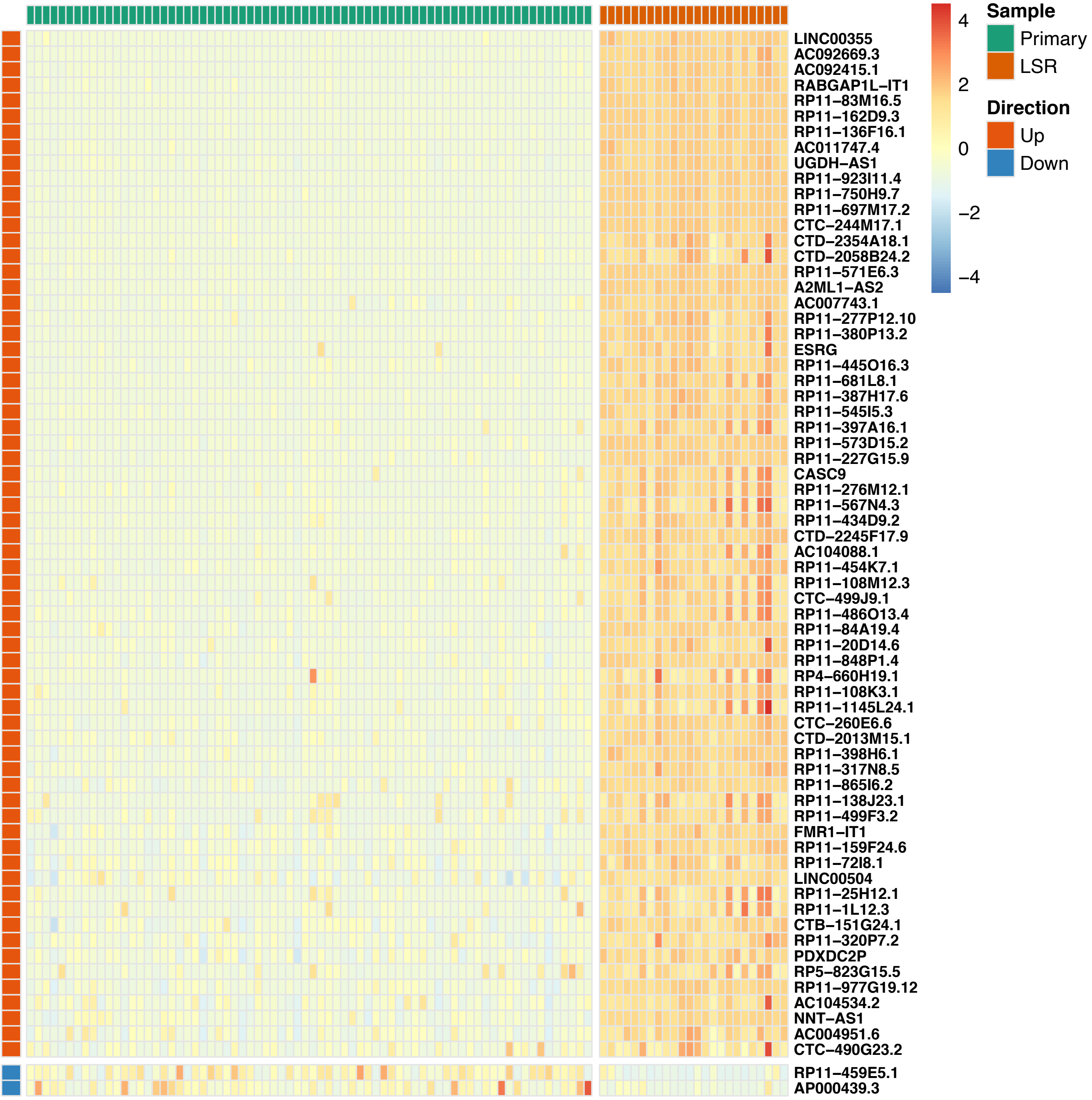

Supplementary Figure 1 Heatmap showing the expression of patient samples for the lncRNAs in late-stage relapse breast cancer. Side bar represents expression (FPKM).

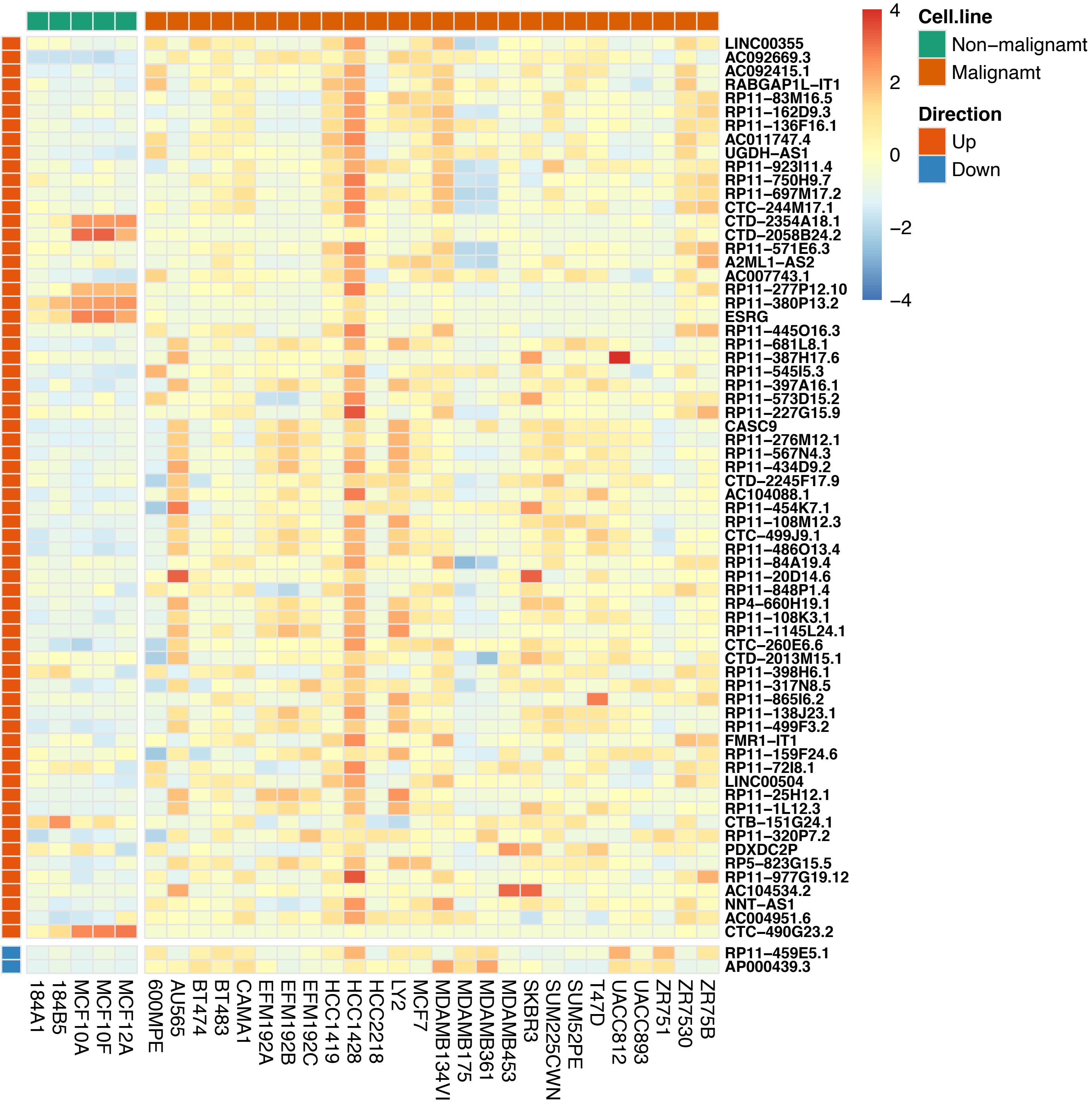

Supplementary Figure 2 Heatmap showing the expression of cell line samples for the lncRNAs in non-malignant and malignant cells. Side bar represents expression (FPKM).

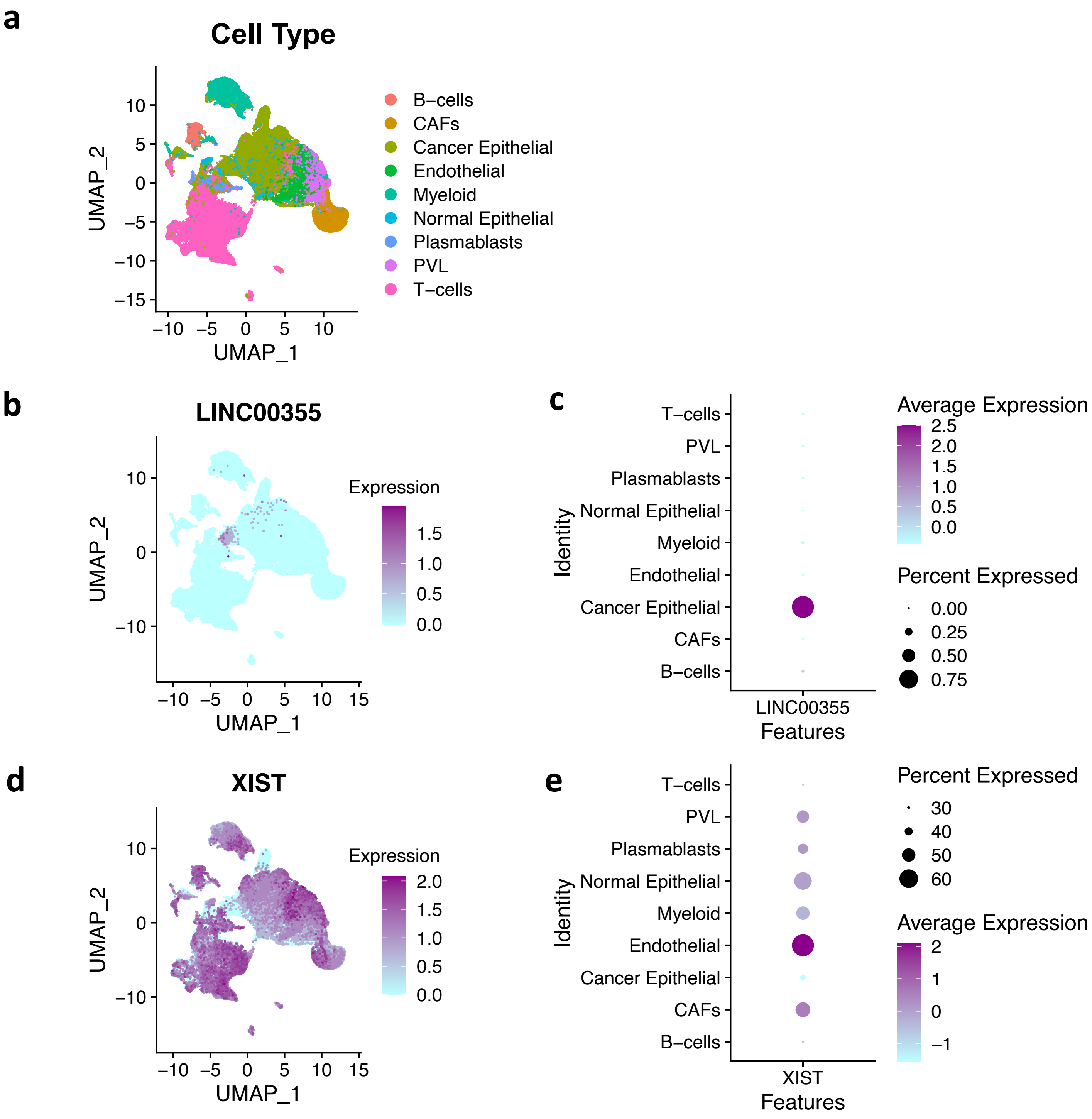

Supplementary Figure 3 (a) Single Cell sequencing data showing cell types from primary breast cancer cells. *LINC00355* and *XIST* expression in breast cancer tissue single cell data showing cell type by UMAP **(b and d)** and by features **(c and e).**

GTEX Bulk Normal Gene expression

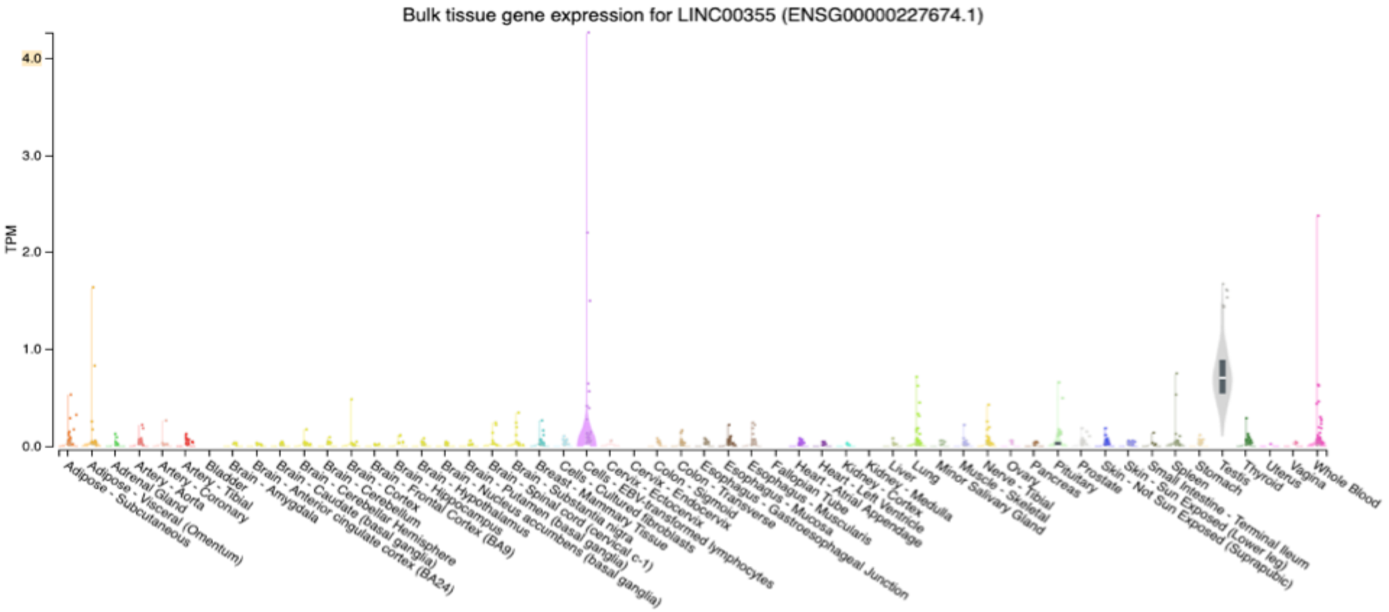

Supplementary Figure 4 *LINC00355* expression in normal tissues from genotype-tissue expression project.

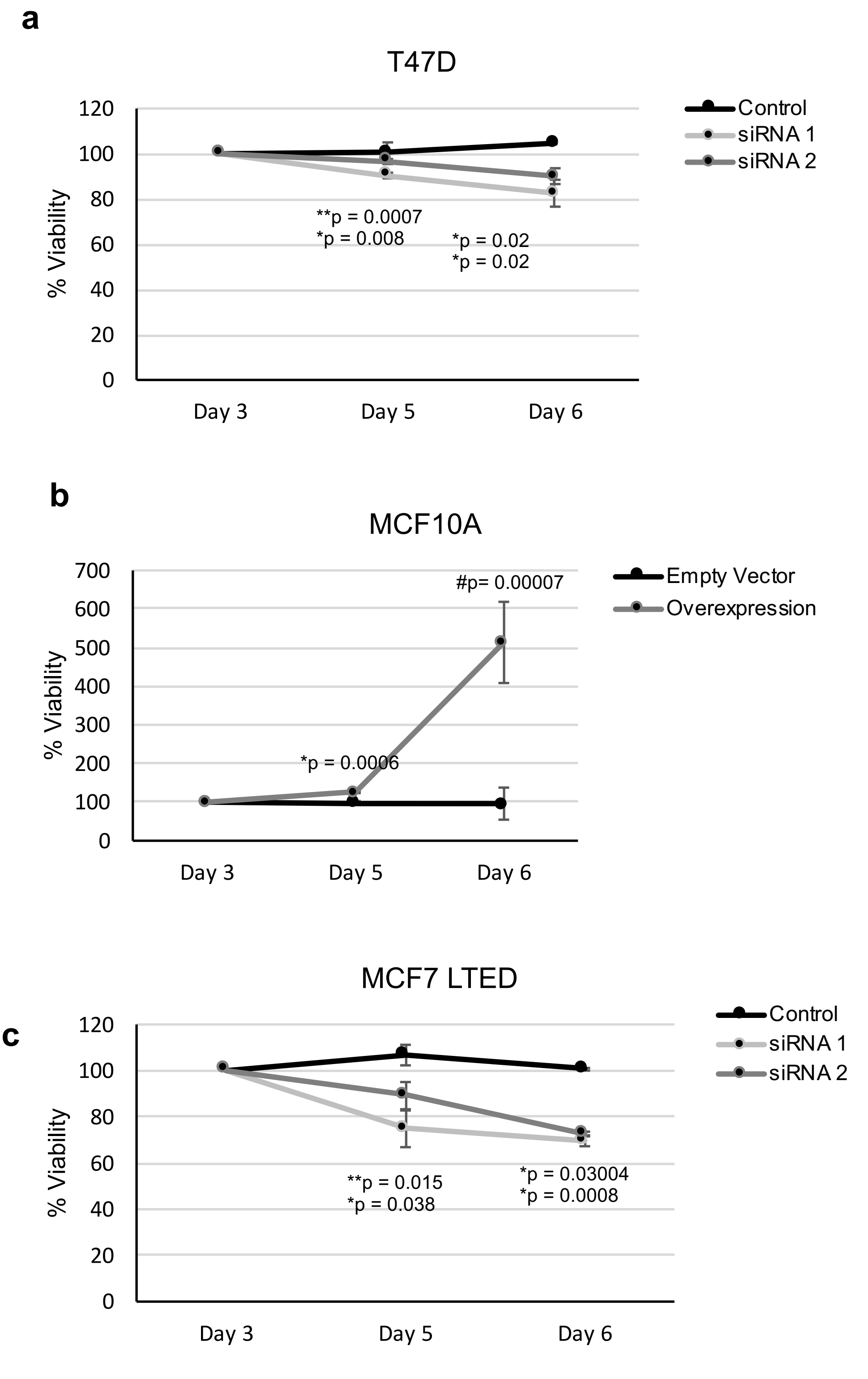

Supplementary Figure 5 (a) Decreased *LINC00355* expression in T47D and MCF7 LTED cells **(c)** decreases proliferation and **(b)** *LINC00355* overexpression in MCF10A cells increases proliferation by Alamar Blue assay. *p value < 0.05, **p value < 0.005, #p value < 0.0005. All data are presented as mean values ± s.d, analyzed by two-tailed paired t-test, and repeated more than two times. Source data are provided as a Source Data File.

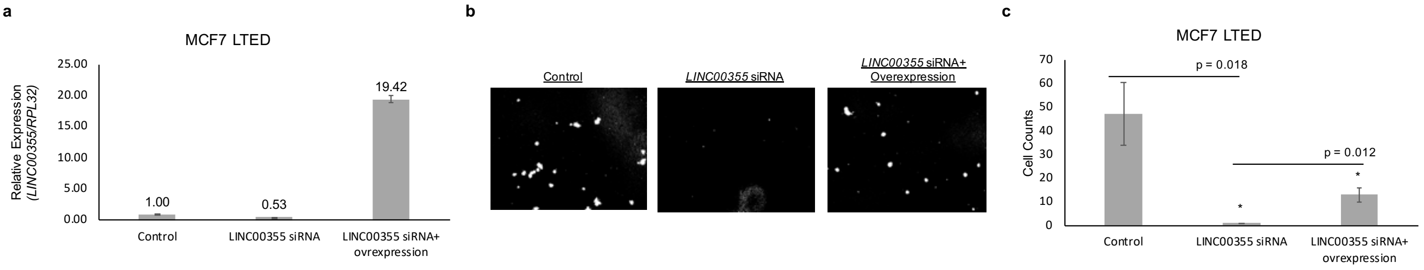

Supplementary Figure 6 (**a**) Transient knockdown and knockdown with subsequent overexpression of *LINC00355* in MCF7 LTED. (**b** and **c**) Cellular invasion decreases with decreased *LINC00355* and increases when *LINC00355* is re-introduced. *p value < 0.05, **p value < 0.005, #p value < 0.0005. All data are presented as mean values ± s.d, analyzed by two-tailed paired t-test, and repeated more than two times.

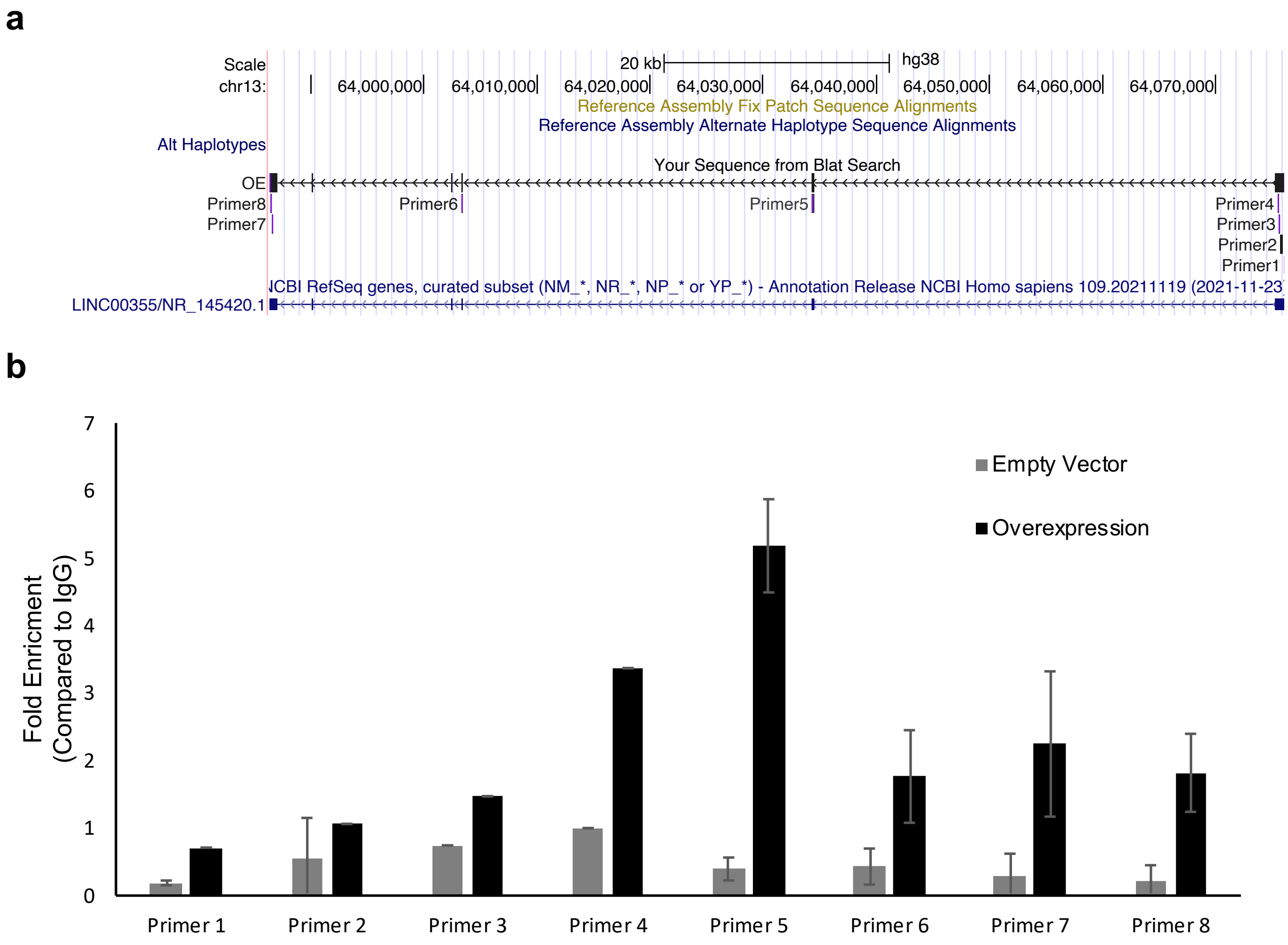

Supplementary Figure 7 (a) UCSC screenshot of sequences for *LINC00355* overexpression (OE) and primers (Primer 1-8) tiling *LINC00355*. **(b)** Cross-linking immunoprecipitation qPCR tiling of MCF10A empty vector and overexpression with MENIN.

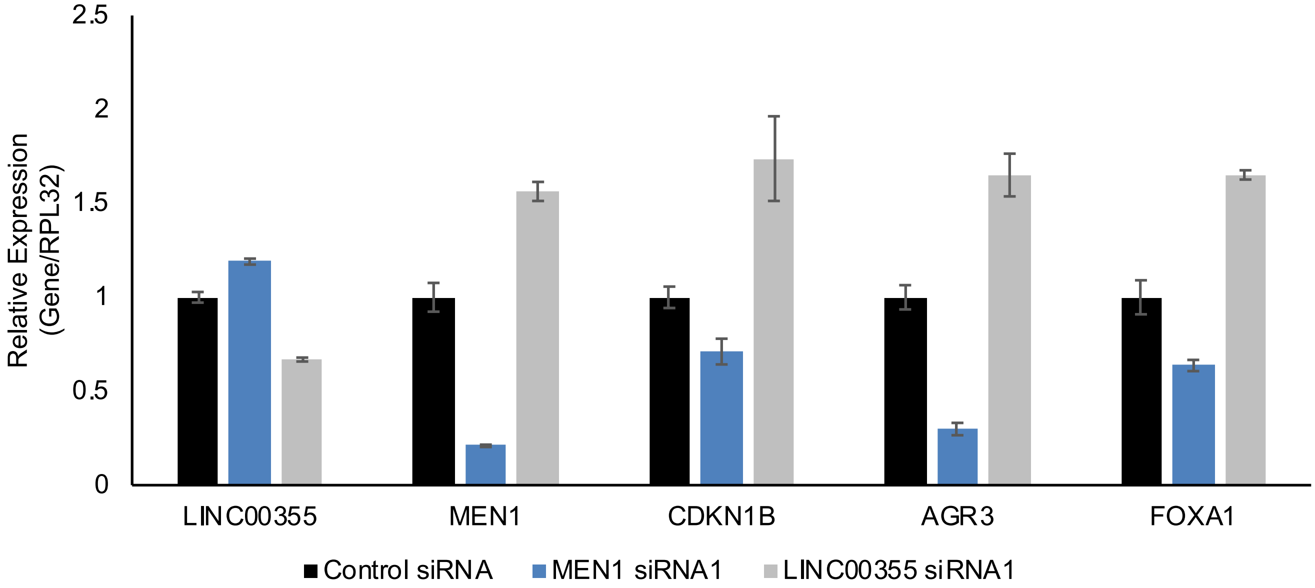

Supplementary Figure 8 (a) Expression of MENIN regulated gene panel with silenced *MEN1*, *LINC00355,* and combination in T47D cells by qPCR.

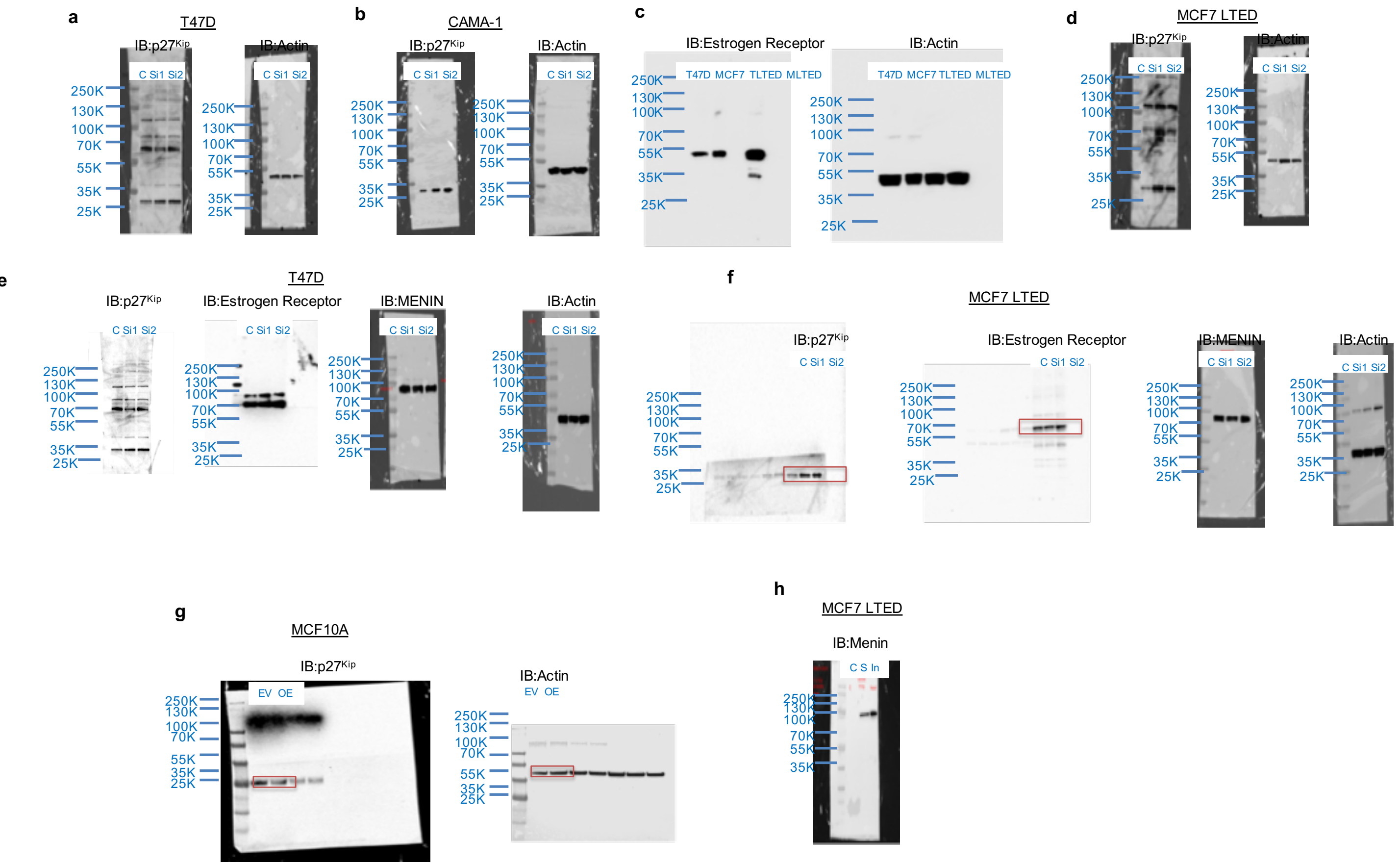

Supplementary Figure 9 Raw blots (a and b) Figure 3e blots **(c)** Figure 4a blots **(d)** Figure 4g blots **(e and f)** Figure 5a blots **(g)** Figure 3l, **(h)** Figure 5 e.
