# Supplementary material for: LINC00355 regulates p27KIP expression by binding to MENIN to induce proliferation in late-stage relapse breast cancer
Source: NPJ Breast Cancer. 2022 Apr 13;8:49. doi: 10.1038/s41523-022-00412-2 (PMC9007952; doi:10.1038/s41523-022-00412-2)
Supplement: Supplementary file 6 — Reporting Summary [file 41523_2022_412_MOESM6_ESM.pdf]

nature portfolio

Corresponding author(s): Jessica M. Silva-Fisher, and Christopher A. Maher

Last updated by author(s): Feb 18, 2022

Reporting Summary

Nature Portfolio wishes to improve the reproducibility of the work that we publish. This form provides structure for consistency and transparency in reporting. For further information on Nature Portfolio policies, see our Editorial Policies and the Editorial Policy Checklist.

Statistics

For all statistical analyses, confirm that the following items are present in the figure legend, table legend, main text, or Methods section.

n/a	Confirmed	
☐	☒	The exact sample size (n) for each experimental group/condition, given as a discrete number and unit of measurement
☐	☒	A statement on whether measurements were taken from distinct samples or whether the same sample was measured repeatedly
☐	☒	The statistical test(s) used AND whether they are one- or two-sided<br>*Only common tests should be described solely by name; describe more complex techniques in the Methods section.*
☐	☒	A description of all covariates tested
☐	☒	A description of any assumptions or corrections, such as tests of normality and adjustment for multiple comparisons
☐	☒	A full description of the statistical parameters including central tendency (e.g. means) or other basic estimates (e.g. regression coefficient) AND variation (e.g. standard deviation) or associated estimates of uncertainty (e.g. confidence intervals)
☐	☒	For null hypothesis testing, the test statistic (e.g. F, t, r) with confidence intervals, effect sizes, degrees of freedom and P value noted<br>*Give P values as exact values whenever suitable.*
☒	☐	For Bayesian analysis, information on the choice of priors and Markov chain Monte Carlo settings
☒	☐	For hierarchical and complex designs, identification of the appropriate level for tests and full reporting of outcomes
☐	☒	Estimates of effect sizes (e.g. Cohen's d, Pearson's r), indicating how they were calculated

Our web collection on statistics for biologists contains articles on many of the points above.

Software and code

Policy information about availability of computer code

Data collection	The primary breast cancer samples and LSR samples were accrued from previous study and at Washington University via dbGAP database and NCBI GEO. Various custom scripts was used for data download. TCGA RNA-Seq data were downloaded the cancer genomics hub and GDC data portal.
Data analysis	All sequencing reads from both patient and cell lines data were aligned to the human reference genome hg19 using TopHat version v2.0.864. Raw read counts for transcripts were generated using featureCounts version v1.4.6-p365 and were used to compute transcript expression levels as normalized in FPKM (Fragments Per Kilobase of transcript per Million mapped reads) format. Differential expression analysis was performed between early-stage and late-stage relapse samples using edgeR version v3.8.666 using the raw read counts. Only transcripts with expression > FPKM in at least 50% of samples in either group were retained for downstream analysis. To correct for batch effect, RUVSeq version v1.0.067 was used. All transcripts with FDR < 0.001 and absolute log fold change > 2 were considered differentially expressed transcripts.

For manuscripts utilizing custom algorithms or software that are central to the research but not yet described in published literature, software must be made available to editors and reviewers. We strongly encourage code deposition in a community repository (e.g. GitHub). See the Nature Portfolio guidelines for submitting code & software for further information.

Data

Policy information about availability of data

All manuscripts must include a data availability statement. This statement should provide the following information, where applicable:

- Accession codes, unique identifiers, or web links for publicly available datasets
- A description of any restrictions on data availability
- For clinical datasets or third party data, please ensure that the statement adheres to our policy

The early-stage breast cancer RNA sequencing data referenced in this study are available from dbGaP database under the accession code phs000472 and the late-stage relapse breast cancer relapse RNA-Seq data generated in this study are available in the NCBI Gene Expression Omnibus (GEO) under the accession code GSE189389. The RNA-Seq data of the breast cancer cell lines referenced in this the study are available in a public repository from the NCBI GEO under the accession code GSE48213. The data used for the GTEx analyses described in this manuscript were obtained from the GTEx Portal (accession number phs000424.vN.pN) on 11/18/21. The source data underlying figures are provided as a Source Data file. All the other data supporting the findings of this study are available within the article, its supplementary information files, and from the corresponding author upon request. A reporting summary for this article is available as a Supplementary Information file.

Field-specific reporting

Please select the one below that is the best fit for your research. If you are not sure, read the appropriate sections before making your selection.

☒ Life sciences ☐ Behavioural & social sciences ☐ Ecological, evolutionary & environmental sciences

For a reference copy of the document with all sections, see nature.com/documents/nr-reporting-summary-flat.pdf

Life sciences study design

All studies must disclose on these points even when the disclosure is negative.

Sample size	Sample size included 72 early stage breast cancer and 24 late -stage relapse breast cancer patient transcriptome sequencing data.
Data exclusions	Experimental assays were excluded if positive or negative controls did not validate.
Replication	qPCR was replicated at least in triplicate to verify knock down of representative genes. Western blots were replicated at least in triplicate. RNA immunoprecipitation and chromatin immunoprecipitation was repeated at least in triplicate. BrU-labeled RNA pulldowns were repeated in at least 2 cell lines. Nuclear cytoplasmic extractions were repeated at least in triplicate in 2-3 different cell lines. Transwell assays were repeated at least in triplicate in multiple cell lines.
Randomization	Randomization was not relevant to this study. We compared specific samples or cohorts to each other.
Blinding	Blinding was not possible in this study as we compared knockdown experiments to each other and need to know which cells were being modified.

Reporting for specific materials, systems and methods

We require information from authors about some types of materials, experimental systems and methods used in many studies. Here, indicate whether each material, system or method listed is relevant to your study. If you are not sure if a list item applies to your research, read the appropriate section before selecting a response.

Materials & experimental systems

n/a	Involved in the study	
☐	☒	Antibodies
☐	☒	Eukaryotic cell lines
☒	☐	Palaeontology and archaeology
☒	☐	Animals and other organisms
☒	☐	Human research participants
☒	☐	Clinical data
☒	☐	Dual use research of concern

Methods

n/a	Involved in the study	
☒	☐	ChIP-seq
☐	☒	Flow cytometry
☒	☐	MRI-based neuroimaging

Antibodies

Antibodies used	p27 (c-19) R Santa Cruz SC-528<br>Anti-menin Abcam ab2605<br>Actin Cell Signaling 3700S

Anti-rabbit HRP linked Cell Signaling 65-6120
Goat anti-mouse HRP-linked Thermo 31430
IgG Cell Signaling 2729S
H3K4me3 Abcam ab12209
Estrogen receptor alpha Cell Signaling D8H8

Validation	Cell Signaling antibodies as indicated by manufacturer are validated to verify specificity, lot-to-lot testing, and isotype controls. Actin manufacture citations are listed here: https://www.citeab.com/antibodies/123338-3700-actin-8h10d10-mouse-mab?utm_campaign=Widget+All+Citations&utm_medium=Widget&utm_source=Cell+Signaling+Technology.<br>IgG manufacture citations listed here:https://www.citeab.com/antibodies/654207-2729-normal-rabbit-igg?utm_campaign=Widget+All+Citations&utm_medium=Widget&utm_source=Cell+Signaling+Technology<br>Estrogen receptor was verified by looking at expression of Er+ cell line<br><br>Activity, stability and performance are important checks carried out by Abcam and Santa Cruz for validation of antibodies (listed on manufacture website).

Eukaryotic cell lines

Policy information about cell lines

Cell line source(s)	All breast cancer cell lines were a kind gift from Dr. Mathew Ellis and Jieya Shao at Washington University in St. Louis (T47D, MCF7, HCC1428, BT483, ZR75B, HCC1500, T47D LTED, MCF7 LTED, CAMA-1, BT-474 cells,MDA175 and MCF10A.
Authentication	Our lab did not authenticate cells lines that were a gift.
Mycoplasma contamination	Cell lines were tested for mycoplasma contamination. If they were positive for mycoplasma we treated cell lines with Invitrogen Plasmocure (cat#ant-pc) for 2 weeks until results came back negative.
Commonly misidentified lines (See ICLAC register)	N/A

Flow Cytometry

Plots

Confirm that:

- [ ] The axis labels state the marker and fluorochrome used (e.g. CD4-FITC).
- [ ] The axis scales are clearly visible. Include numbers along axes only for bottom left plot of group (a 'group' is an analysis of identical markers).
- [ ] All plots are contour plots with outliers or pseudocolor plots.
- [x] A numerical value for number of cells or percentage (with statistics) is provided.

Methodology

Sample preparation	T47D, CAMA1, MCF10A, MCF7-LTED were used for flow cytometry.
Instrument	Becton Dickinson FACScan
Software	FlowJo software v10 was used for data collection and analysis.
Cell population abundance	25,000 cells were assessed and greater than 97% live single cells were used for each cell population.
Gating strategy	Greater than 97% live cells FSC/SSC gates were used for each starting cell population.

- [ ] Tick this box to confirm that a figure exemplifying the gating strategy is provided in the Supplementary Information.
